# Importance of lactate dehydrogenase (LDH) and monocarboxylate transporters (MCTs) in cancer cells

**DOI:** 10.1002/hsr2.996

**Published:** 2022-12-21

**Authors:** Hamed Hatami, Atefe Sajedi, Seyed Mostafa Mir, Mohammad Yousef Memar

**Affiliations:** ^1^ Department of Immunology, Faculty of Medicine Mashhad University of Medical Sciences Mashhad Iran; ^2^ Metabolic Disorders Research Center Golestan University of Medical Sciences Gorgan Iran; ^3^ Department of Clinical Biochemistry, Faculty of Medicine Golestan University of Medical Sciences Gorgan Iran; ^4^ Infectious and Tropical Diseases Research Center Tabriz University of Medical Sciences Tabriz Iran

**Keywords:** cancer metabolism, lactate, monocarboxylate transporters

## Abstract

**Background:**

In most regions, cancer ranks the second most frequent cause of death following cardiovascular disorders.

**Aim:**

In this article, we review the various aspects of glycolysis with a focus on types of MCTs and the importance of lactate in cancer cells.

**Results and Discussion:**

Metabolic changes are one of the first and most important alterations in cancer cells. Cancer cells use different pathways to survive, energy generation, growth, and proliferation compared to normal cells. The increase in glycolysis, which produces substances such as lactate and pyruvate, has an important role in metastases and invasion of cancer cells. Two important cellular proteins that play a role in the production and transport of lactate include lactate dehydrogenase and monocarboxylate transporters (MCTs). These molecules by their various isoforms and different tissue distribution help to escape the immune system and expansion of cancer cells under different conditions.

## 1 INTRODUCTION

There is a significant increase in the incidence and prevalence of noncommunicable diseases, despite many successful advances in controlling and preventing contagious diseases in recent decades.^1^, ^2^, ^3^ Meanwhile, cancer is the second leading cause of mortality after cardiovascular diseases in some countries^4^, ^5^ and is one of the principal causes of death throughout the world, which is heterogeneous from the aspect of genetically. Despite different genetic variations, the common feature of all cancer types is their excessive growth.^6^, ^7^ One of the most remarkable changes of cancer cells is altering their metabolism, which has recently been considered as an important and effective factor in the progression of cancer.^8^, ^9^ Previous studies have shown that we can inhibit the tumor cells growth and survival throughout halting or reducing their cellular metabolism.^10^, ^11^ Changing lactate metabolism as an anaerobic glycolysis by‐product that mainly produced in the absence of oxygen in the body is one of the adaptive proceedings of tumor cells for survival and further progression.^8^ Tumor early development and growth occur in the low vascular regions. There are two different cell types in the tumor lesions included aerobic region and hypoxic regions. Aerobic region included the cells that reside near blood vessels and their energy demands provided through oxidative phosphorylation (OXPHOS), while hypoxic regions are cells that reside far from blood vessels and their energy demands provided through the anaerobic metabolism of glucose. The metabolism of cancer cells has considered equivalent to aerobic glycolysis for a long time. After a while, this theory has been considered imperfect by biologists. The cancer cells metabolic signatures not only do not result in passive response to mitochondrial damage but also because of the reprogramming of the metabolism of cancer cells arose from oncogenes activity needed for anabolic growth.^12^ Progresses in cancer metabolism studies over the last year have improved our knowledge of how aerobic glycolysis and other metabolic changes detected in cancer cells provide the anabolic needs related two cell proliferation and development. Understanding the mechanisms employed by cancer cell to metabolism and survival can lead to the development of new strategies in fighting cancers and increase our understanding on the behavior of cancer cells. In this article, we review the various aspects of glycolysis with a focus on types of monocarboxylate transporters (MCTs) and the importance of lactate in cancer cells.

## 2 METHODOLOGY

In this study, data on various aspects of glycolysis with a focus on types of MCTs and lactate in cancer cells were found using PubMed, Scopus, and the Google Scholar database. The Internet searches were done to find published manuscripts with the keywords Cancer metabolism, Monocarboxylate transporters, lactate. All English language articles were found and read independently by two individuals. Literature search strategy for data in tables including inclusion/exclusion criteria and results was provided in Supporting Information: Figure S1.

## 3 RESULTS AND DISCUSSION

### 3.1 Advantages of aerobic glycolysis pathway for tumor growth

Even in the presence of ample oxygen, cancer cells demonstrate a distinctive form of cellular metabolism characterized by high levels of glucose uptake and increased conversion of glucose to lactose via the glycolytic pathway (fermentation). This phenomenon called the Warburg effect and known anaerobic glycolysis, has been recognized for many years. Warburg metabolism is not cancer‐specific, but instead is a general property of growing cells that is exploited by cancer cells. Aerobic glycolysis provides rapidly dividing tumor cells with metabolic intermediates that are needed for the synthesis of cellular components, whereas mitochondrial OXPHOS does not.

The aerobic glycolysis pathway provides several advantages for tumor cells growth as below. Using aerobic glycolysis, the cancerous cell can survive in variable levels of oxygen pressure (due to unstable hemodynamics of blood vessels) and produce ATP (this condition is associated with risk of death for OXPHOS‐dependent cells).^13^

Tumor cells produce bicarbonate and lactate. These acids make the microenvironment suitable for invasive tumor cells and suppress the immune system.^14^, ^15^, ^16^ Lactate produced by tumor cells can be uptake by stromal cells (by Monocarboxylate transporter 1 [MCT1] and MCT2) and used to pyruvate produce.^15^

The most important advantage is using the glycolysis‐pathway mediators for anabolic reactions (e.g., use of glucose 6‐phosphate for the synthesis of glycogen and ribose 5‐ phosphate and nicotinamide adenine dinucleotide phosphate [NADPH], dihydroxyacetone phosphate for the synthesis of triglycerides and phospholipid, pyruvate for the synthesis of alanine and malate).^17^ The exploitation of the pentose phosphate pathway (PPP) as one of the glycolysis branching commonly increases in tumorgenesis. Moreover, some of the major enzymes of this pathway, such as transketolase 1 and transaldolase, have shown increased expression in many cancers.^18^, ^19^, ^20^, ^21^ Also, one of the reasons for reducing the use of the Krebs cycle is some of the products of this cycle, such as nicotinamide adenine dinucleotide hydrogen (NADH) and adenosine triphosphate (ATP), which are the main inhibitors of glucose metabolism.^22^

### 3.2 Metabolic changes in cancer cells

#### 3.2.1 Absorption of glucose and amino acids (increased demand for nitrogen)

Glucose and glutamine (unnecessary amino acid with two reduced nitrogen atoms) are the most important and essential precursors for the biosynthesis of cellular materials). Due to increased carbon consumption in biosynthetic pathways and growth signals, the cell's need for nitrogen increases. Transcription factors such as C‐MYC and E2F increase glutamine uptake.^22^ Lack of glutamine leads to cell cycle arrest in the S‐phase in some cells.^23^, ^24^ Embryonic stem cells, luminal cells of breast cancer,^25^, ^26^ and human glioblastoma tumors continue their growth and proliferation, even in the absence of glutamine in their microenvironment, which represents the glutamine production by these cells de novo.^27^ It also has been seen an increase in the expression of glutamine synthase (GS) enzyme in some cancers.^28^

#### 3.2.2 Opportunity to absorb nutrients

The Ras or c‐Src mutation enables cells to uptake released amino acids by the lysosomal degradation of extracellular proteins.^29^ Macropinocytosis is also stimulated by Ras‐ and c‐Src‐driven actin cytoskeleton remodeling.^22^ Moreover, soluble extracellular proteins and free amino acids can be absorbed into cancer cells by entosis or phagocytosis.^30^, ^31^

Cancer cells, in addition to the absorption of fatty acids from plasma, can induce the release of stored lipids in adjacent normal cells.^22^ Furthermore, increased expression of monoacylglycerol lipase (MAGL) and lipoprotein lipase in some of the cancer cells has been related with the invasion of them.^32^, ^33^ At the surface of the metastatic ovarian cancer cells, the expression changes of fatty acid binding protein 4 (FABP4) enable these cells to absorb fatty acids from the omental fat adipocytes.^34^ However, fatty acid de novo biosynthesis in normal cells is very low (except lipogenic tissues such as the liver, adipose tissue, and mammary epithelium during lactation).^35^, ^36^

#### 3.2.3 Metabolic interactions with the environment

The increase in reactive oxygen species (ROS) production (due to OXPHOS pathway and excessive ATP production, cellular degradation, cellular overgrowth, and oncogene‐induced senescence [OIS]),^22^ inhibit protein phosphatases such as phosphatase and tensin homolog (PTEN), protein tyrosine phosphatase 1B (PTP1B) and activators of family kinases of Src and mitogen‐activated protein kinase (MAPK).^37^, ^38^, ^39^ Other effects of enhancement of ROS are facilitating the activation of hypoxia‐inducible factor 1‐alpha (HIF1‐α) and nuclear factor erythroid 2‐related factor 2 (NRF2).^40^, ^41^ In hypoxia conditions, induced overexpression of mitochondrial SHMT2 (serine hydroxyl methyl transferase 2) protects cells against the toxic effects of oxidative stress.^42^

**Figure 1:**
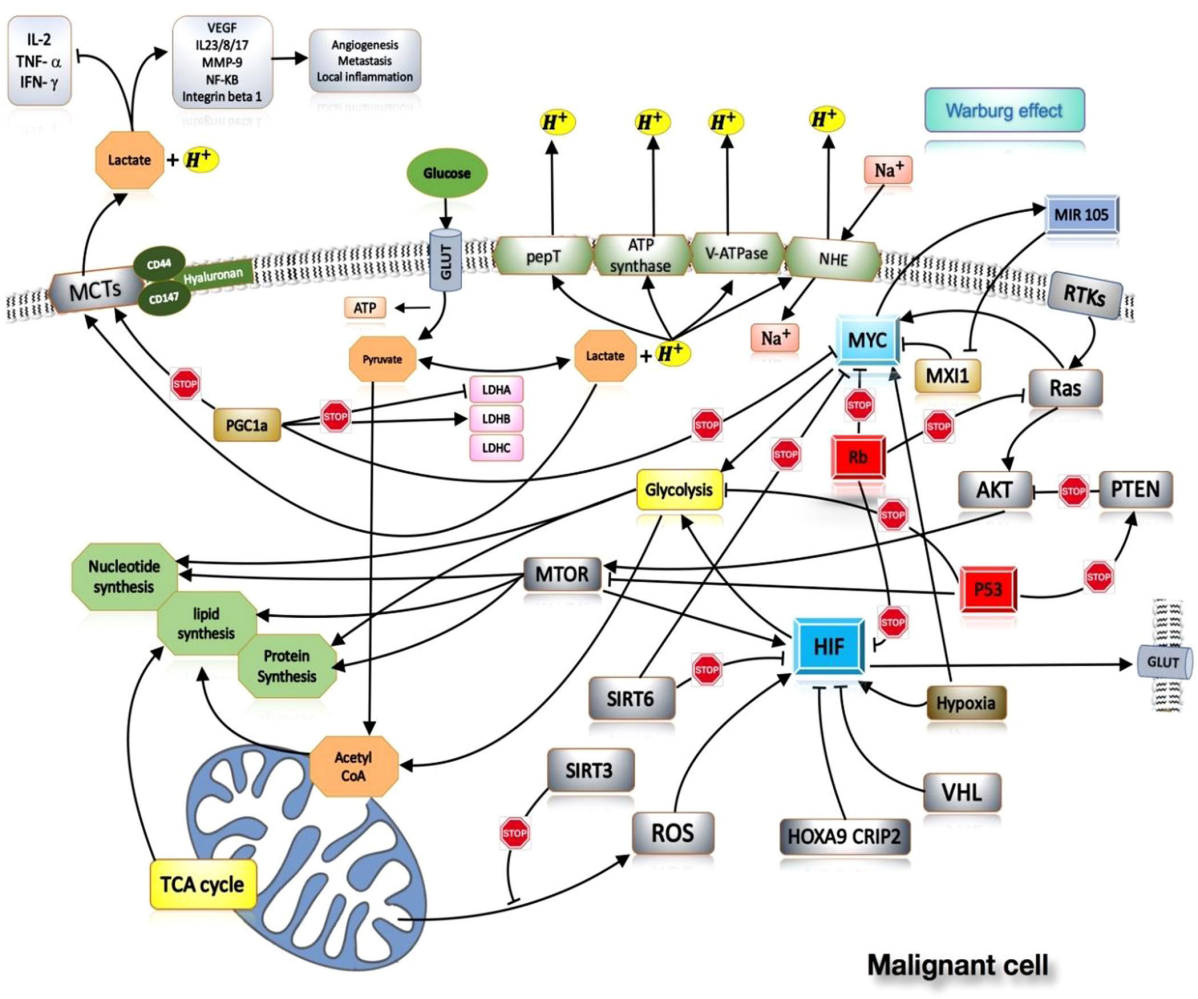
The metabolic changes in the malignant cells. After conversion of glucose to pyruvate and entrance into the oxidative phosphorylation pathway, pyruvate converts to acetyl CoA. Acetyl CoA by entering the mitochondria (Krebs cycle) generates energy. In most of the malignant cells, after pyruvate generation, these cells use the aerobic glycolysis pathway (Warburg effect (pyruvate by lactate dehydrogenase, LDH, enzymes convert into lactate and proton)). Produced lactate has been sent out of the cell by monocarboxylate transporters (MCTs) and has been shown that play important role in increases the angiogenesis, migration, and metastasis of cells, escape from the immune system, extracellular acidosis, reduction of the monocytes migration, secretion of cytokines and activation of T lymphocytes. Moreover, with the help of these monocarboxylates transporter, lactate can reenter to the cell and again converts to pyruvate by the LDH and generates energy. With the increase in glycolysis, proton production also increases, and these excess protons through proton pumps such as MCT, H/Na exchanger, ATP synthase, pepT, V‐ATPase are transmitted into extracellular space and causes acidosis in the microenvironment of the cancer cells. Activation of oncogenes, such as MYC, HIF, RAS, and Akt, and interaction of them with different molecules in the cell, is also the major cause of the biogenic changes in tumor cells.

The increase in lactate production in cancer cells has several effects on the other host tissue and cells. Many studies highlighted the effects of lactate in suppressing on the immune cells such as activation of dendritic cell and T cell, migration of monocytes, and stimulation of M2 macrophage. M2 macrophages play an important role in the inhibition of the immune system and wound healing.^14^, ^43^, ^44^, ^45^, ^46^ Several other effects have been described for lactate on the host body cells such as induction of angiogenesis,^47^ induction of HIF1a stability,^48^ activating of nuclear factor kappa‐light‐chain‐enhancer of activated B cells and PI‐3k signaling pathways,^49^ triggering the secretion of angiogenesis factors such as vascular endothelial growth factors (VEGFs)^47^, ^50^, ^51^, ^52^ stimulate and stimulating the production of acid hyaluronic by fibroblasts (may play a role in cellular invasiveness).^53^ Some important factors involved in tumor cell metabolism and their effects listed in Table 1 and Figure 1.

**Table 1:** Some factors involved in the metabolism of tumor cells and their effects

Factor	Effect	References		
SREBP	Stimulating the expression of genes involved in fatty acid and sterol biosynthesis Regulate the cell growth and increase it Stimulated by PKB/Akt and MTOR pathways Interaction with FBI‐1	[143, 144, 145, 146]		
FBI‐1 (Pokemon/ZBTB7A)	Increasing of SREBP activation and cell growth	[143]		
MYC (Figure 1)	Increasing of the expression of genes involved in the uptake and consumption of glucose, such as glycolysis enzymes Increasing of glutamine consumption Stimulating the expression of enzymes involved in the synthesis of fatty acids and lipids Stimulating the expression of enzymes involved in anabolic pathways such as glycine and serine metabolism Increase the expression of mitochondrial genes and its biogenesis Helping to the growth and invasion of cancer cells Interaction with beta‐catenin/TCF signaling pathway and increasing MCT1 and PDK1 genes regulation and transcription Regulation of primary enzymes of purine and pyrimidine biosynthesis Facilitating glutamine reabsorption In cancer cells, increases the production of mir‐105, which is transfer by exosome. Mir‐105 reduces the level of MYC inhibitor transcription (MXI1) levels, thereby increasing the expression of MYC‐dependent genes	[147, 148, 149, 150, 151, 152, 153, 154]		
mTORC1	Activating of HIF1 Increase the translation of cyclin D1 and c‐Myc and reduce the translation of inhibitors such as cyclin‐dependent kinase inhibitors and p27 Aerobic glycosis regulation Play a role in cellular growth (with the cooperation of eIF4B and S6K subunits) and increasation protein synthesis Antiapoptotic activity (with the cooperation of the eIF4E subunit)	[155, 156, 157]		
HIF1 (Figure 1)	Increasing of glucose consumption Increasing of lactate and ATP production without oxygen dependency The induction of angiogenesis in tumor cells by activation and regulation of the genes expression such as VEGF, Ang‐1, Ang‐2 Increasing of the survival and proliferation of tumor cells by activation and regulation the expression of IGF2 gene and activating the MAPK and PI3K signaling pathway	[7, 158]		
PI3K	Activating the Akt signaling pathway Decrease in expression of CPT1A (carnitine palmitoyltransferase) enzyme Changing in this pathway is one of the most common events in human cancers Increasing of glucose uptake, lipid synthesis, and glycolysis	[159]		
Akt (Figure 1)	Increase in regulation of fatty acid production Increasing of expression of FAS enzyme Activating of mTOR pathway Increase cell growth and invasion	[160]		
ErbB2 (Her2)	Increasing the synthesis of fatty acid and cell growth (by increasing the regulation of acetyl‐CoA carboxylase alpha and fatty acid synthase) mTOR, Akt, and PI3K signaling pathways activating	[161]		
PD‐L1	Its expression regulated by oncogenic transcription factors, such as C‐MYC and HIF In cancer cells of the ovary and melanoma, the high expression of PD‐L1 promotes cell growth and autophagy through the mTOR pathway. The PD‐L1 connects directly to ITGB4 and activates the AKT/GSK3β and the ITGB4/1/SNAI3 SIRT signaling pathways The high expression of PD‐L1 and ITGB4 in cervical cancer was significantly associated with metastasis and poor prognosis of lymph nodes	[162, 163, 164, 165, 166, 167, 168, 169, 170, 171, 172]		
CAV1/caveolin 1	Increasing of aerobic glycolysis (by SLC2A3/GLUT3 activating)	[173, 174]		
CD147 (Figure 1)	Stimulated by HIF‐1α and Sp1 Inducing of glycolysis and role in development, proliferation, invasion, and metastasis of tumor cells	[175, 176]		
Ecdysoneless	Glycolysis regulation and role in metastasis and proliferation of tumor cells	[177]		
FAK	Glucose uptake stimulation and shifting its consumption from oxidative phosphorylation to glycolysis Glycolysis and tumorgenesis induction	[178]		
GRP78	Stimulated by HIF‐1a Stimulation of glucose Consumption, angiogenesis, and metastasis Induction of autophagy, resistance to apoptosis, and escape from the immune system Reduction of glycolysis and shifting glucose consumption from glycolysis to Krebs cycle Decrease the expression of PKM2 and increase the expression of mitochondrial PDHA and B PDHB and Glut‐1	[179]		
KSHV	Induction of aerobic glycolysis and angiogenesis by HIF‐1a and PKM2 Increase in VEGF levels	[180]		
LMP1	Stimulation of aerobic glycolysis, cell growth, metastasis, invasion, and angiogenesis Increasing of glucose and glutamine absorption and lactate production Increase in expression of genes such as PDHK1, FGFR1, c‐Myc, HIF‐1α	[181]		
P2X7R	Inhibition of pyruvate dehydrogenase Akt/PKB and HIF‐1α pathways inducing Increasing of glycogen intracellular storage Increase regulation of Glut1, G3PDH, PFK, PKM2, and PDHK1	[182]		
Skp2	Akt activation Role in metastasis, poor prognosis, and disease progression Increase in glucose uptake and consumption (by Glut1 expression increasing)	[183]		
BIRC5/Survivin	Increase aerobic glycolysis and drug resistance Apoptosis inhibition (BCL2L11/Bim inhibition) Reduced mitochondrial respiration	[184]		
Wnt/β‐catenin	Increase in aerobic glycolysis, angiogenesis, and cell growth	[152]		
p53 (Figure 1)	Role in glucose and fatty acids metabolism (inhibition of glycolysis and glucose transporters, negative regulation of phosphoglycerate mutase and AKT) Stimulation of the catabolic pathways (i.e., fatty acids oxidation) Increase ROS production	[185, 186, 187, 188]		
Fumarate hydratase (fumarase)	Helping to HIF‐1a degradation (Fumarate accumulation inhibits HIF1a prolyl hydroxylase)	[189]		
*abhd5* (other name: CGI‐58)	Cell growth reduction inhibition aerobic glycolysis and EMT The AMPKα‐p53 pathway inducing	[190]		
GRIM‐19	Aerobic glycolysis reducing Decreasing of cell growth and proliferation Increase P53 activity Decrease Stat3 and HIF‐1a activity Increase oxygen consumption	[191]		
HSP40	Binds to pyruvate kinase M2 and destroy it that resulted to reducing glucose consumption and lactate production Reduce growth and cell proliferation	[192]		
KLF4	Reducing of aerobic glycolysis and decreasing of cell growth and proliferation LDHA expression reduction	[193]		
TRAP1	Decrease mitochondrial respiration and oxidation of fatty acids Decrease cell growth and invasion Decrease production of ATP and ROS	[194]		
FBI‐1 (also known as Pokemon, ZBTB7A)	Inhibition of glycolysis genes such as PFKP, PKM, GLUT3 Increasing of SREBP activation and cell growth	[143, 195]		

Abbreviations: abhd5, α/β‐hydrolase domain‐containing 5; Ang‐1, angiopoietin 1; Ang‐2, angiopoietin 2; EMT, epithelial‐mesenchymal transition; FAK, focal adhesion kinase; FAS, fatty acid synthetase; FBI‐1, factor that binds to the inducer of short transcripts of human immunodeficiency virus‐1; FGFR1, fibroblast growth factor receptor 1; G3PDH. glyceraldehyde 3‐phosphate dehydrogenase; GRIM‐19, gene associated with retinoid‐interferon‐induced mortality‐19; GRP78, glucose‐regulated protein 78; HIF, hypoxia‐inducible factor; HSP40, heat shock protein 40; IGF2, insulin‐like growth factor 2; ITGB4, integrin β 4; KLF4, Krüppel‐like factor 4; KSHV, Kaposi's‐sarcoma‐associated herpesvirus; LDHA, lactate dehydrogenase A; LMP1, latent membrane protein 1; MAPK, mitogen‐activated protein kinase; MCT1, monocarboxylate transporter 1; P2X7R, P2X7 receptor; PDHA, pyruvate dehydrogenase A; PDHB, pyruvate dehydrogenase B; PDHK1, pyruvate dehydrogenase kinase 1; PI3K, Phosphoinositide 3‐kinase; PKB, protein kinase B; PFK, phosphofructokinase; PKM2, pyruvate kinase M2; ROS, reactive oxygen species; SIRT, selective internal radiation therapy; Sp1, specificity protein 1; SRE, sterol‐responsive element; SREBP, SRE‐binding protein 1; TRAP1, TNF receptor‐associated protein; VEGF, vascular endothelial growth factor.

### 3.3 Glycolysis pathway and cancer cells

The result of the anaerobic metabolism of glucose in cancer cells that reside far from vessels is lactate production, which releases from these cells and entered into the intercellular spaces. This metabolite is entered to near vasculature cancer cells from intercellular space and provides their energy supply.^9^

The glycolysis pathway in cancer cells allows them to consume most of the cell glucose storage and convert it to lactate. Lactate stimulates and enhances cancer cell growth and proliferation. Therefore, it is imperative to recognize and survey the genes involved in this pathway, especially the genes that their product plays an important role in lactate metabolism.^54^ For example, some cancer cells such as cells in glioblastoma use aerobic glycolysis pathway for energy production even in the presence of oxygen (Warburg effect) (Figure 1).^55^

As noted earlier, a large quantity of lactate and proton is produced due to the increasing glycolysis. This extra proton led to an acidic space inside the cell, which can induce normal cell apoptosis under normal conditions.^56^ However, this extra proton is sent out or used by cancer cells through different mechanisms^57^ such as using the family of MCTs,^58^ H/Na exchanger (NHE),^59^ V‐ATPase pump,^60^, ^61^ carbonate anhydrase enzyme,^16^ and the conversion of glutamate to gamma aminobutyrate.^62^ Since this acidity increases the cancer cells invasion potentials as well as drug resistance; therefore, these molecules are overexpressed in cancerous cells than normal cells.^55^ Moreover, not only lactate acts as a signal to stimulate angiogenesis but also is an immunosuppressive agent.^63^

There are two main checkpoints for regulating the production and transport of lactate in the cell included lactate dehydrogenase (LDH) and the family of MCTs. LDH has an important role in the conversion of pyruvate to lactate, and MCTs are involved in the transport of lactate into and out of the cells.^8^ These monocarboxcylates, like lactate, can be transferred to other cancer cells, or use for OXPHOS.

#### 3.3.1 LDH

LDH is produced in normal cells such as the brain, heart, liver, kidney, skeletal muscle, red blood cells, and lungs.^64^, ^65^ However, LDH is overexpressed in some conditions such as hypothyroidism, anemia, meningitis, myocardial infarction, acute pancreatitis, acquired immunodeficiency syndrome (AIDS), liver/pulmonary disease, as well as in most of cancerous cells. Therefore, it can be used as a cancer cell marker or as a therapeutic option for cancer.^66^

This enzyme is found in different organisms. LDH converts pyruvate to lactate when oxygen is absent or trace and it performs the reverse reaction in the Cori cycle in the liver.^64^ Therefore, LDH is an enzyme with two forward and reverse functions. Structurally, this enzyme is a tetrameric enzyme composed of two major subunits (A, B), which are encoded by two the Lactate dehydrogenase A (*LDHA*) (11p15) and *LDHB* (12p12) genes. These two subunits produce five isoenzymes.^65^, ^67^ Peroxisome proliferator‐activated receptor‐γ coactivator 1α (PGC‐1α) by reducing the mRNA transcriptions of the *LDHA* and *MYC* genes and increasing the mRNA transcriptions of the *LDHB* and *MCT1* genes regulate the conversion of pyruvate to lactate gene^68^ (Figure 1).

#### 3.3.2 MCTs

Monocarboxylate is referred to the compounds such as lactate, pyruvate, ketone bodies, and thyroid hormones that play an important role in the energy metabolism of different tissues (i.e., skeletal muscle, heart, brain, and blood cells).^69^, ^70^ Among monocarboxylates, lactate is considered as one of the most important glycolysis products. MCTs facilitate the transport of lactate and other monocarboxylates, thus have an important role in cellular metabolism.^71^

This family of monocarboxylic transporters (MCTs) is considered as one component of the SLC16 family. These soluble carriers structurally consist of 12 transmembranes domain with a cytoplasmic C‐terminal and N‐terminus and one intracellular loop between domain 6 and 7.^72^, ^73^ MCTs in their transmembrane domains have the most conservative sequence, especially in the transmembrane one and five domains. However, in N and C terminal regions more diversity was seen.^74^ This family constitutes of 14 members that are identified by similar conserved amino acid sequence and often they are different in the type of substrate and their tissue distribution (Table 2). The first four members of this family (MCT1‐4) are proton‐linked transporter that facilitate transporting of short‐chain monocarboxylates such as lactate, pyruvate, butyrate, and ketone bodies, and another this family members are involved in the transport of sodium‐coupled monocarboxylic (SMCT) such as thyroid hormones.^71^ More precisely, MCT1, 2, 4 is involved in the transport of lactate, pyruvate, butyrate, acetate, and beta‐hydroxybutyrate. MCT3 plays a role in the transport of lactate.^73^, ^75^ MCT5‐14 transfer certain substances,^76^ for example, MCT9 in the transfer of carnitine and hemostasis of urate,^76^, ^77^ MCT8‐10 in the transfer of thyroid hormones,^73^ and MCT 10 in the transfer of aromatic amino acids,^78^, ^79^ MCT 12 in the transfer of creatine,^80^ and guanidine acetate homeostasis,^76^ and MCT6 in the transfer of xenobiotic compounds such as bumetanide, nateglinide, and probenecid have an important role.^76^

**Table 2:** MCT types and their features

Protein	Gene name and locus	Subcategories	Properties	Tissue distribution	References		
MCT‐1	SLC16A1 1p13.2	MOT1 MEV MCT1D HHF7	Specific substrates (I) Short‐chain monocarboxylate such as halides, hydroxyl and carbonyl groups (II) Short‐chain unsaturated fatty acids such as acetate, propionate and butyrate (III) l‐Lactate, β‐hydroxybutyrate, acetoacetate (IV) Phenylbutyrate (from phenylalanine), keto isocaproat (from leucine), keto iso valerate (from valine) and ketone beta methyl valerate (isoleucine) Inhibitors CHC analogues, sodium sulfonate, (DIDS), (DBDS), (pCMBS), AR‐C155858, amphiphilic compounds include bioflavonoids (e.g., quercetin and fluortine), anion transport inhibitors such as 5‐nitro‐2 benzoate and niflumic acid, AZD3965 Expression enhancing agents (1) Interaction with intracellular carbonic anhydrase II (2) Chronic stimulation or exercise (3) Increasing calcium and cAMP by activating protein phosphatase, calcineurin, and AMPK (4) Cyclosporine A (5) Activation and proliferation of T lymphocytes (6) PGC1a (7) Left ventricular hypertrophy (8) Obesity (9) Localized ischemic (10) AICAR, an AMPK activator (11) Thyroid hormone (T3) (12) Cobalt and hypoxia (13) Promoter demethylation (14) P53 defects (15) Inappropriate expression of MCT1 results in a signaling cascade stimulating ATP synthesis and insulin secretion Expression decreasing agents: Nervous disruption or spinal cord injury	Most tissues except pancreatic β cells	[72, 75, 119, 196, 197, 198, 199, 200, 201, 202, 203]		
MCT‐2	SLC16A7 12q14	MOT2	(1) 50% sequence similarity with MCT8 (2) 60% Homology with MCT1 (3) Cotransporter/H+ Inhibitors (1) AR‐C155858 (2) CHC, DBDS, and DIDS, AZD3965 (3) α‐cyano‐4‐hydroxycinnamate, anion‐channel inhibitors, and flavonoids Expression enhancing agents (1) interaction with extracellular carboxylic anhydrase IV (2) Insulin and IGF‐1 (3) Brain‐derived neurotrophic factor (BDNF) (4) Deprivation of food (48 h) (5) Obesity (6) Localized ischemic (7) Stimulate paths PI3K‐Akt–mTOR (8) Promoter demethylation Expression decreasing agents Hypoxia	Liver, kidney, spleen, heart, brain, testis, pancreas	[72, 75, 119, 197, 199, 204, 205, 206, 207, 208]		
MCT‐3	SLC16A8 22q13	REMP	(1) Respectively 43% and 45% sequence similarity with MCT2, MCT1 (2) Cotransporter/H+	Eye	[74, 197]		
MCT‐4	SLC16A3 17q25.3	MCT3	(1) 55% sequence similarity with MCT3 (2) 44% sequence similarity with MCT2 (3) Inhibition by DIDS or CHC (4) Cotransporter/H+ Expression enhancing agents (1) Interaction with intracellular carbonic anhydrase II (2) Obesity (3) Thyroid hormone (T3) (4) In response to stimulating AMPK with AICAR (5) Hypoxia Promoter demethylation	Heart, brain, skeletal muscle, lung, placenta, kidney, leukocyte, chondrocytes, testis	[72, 73, 75, 119, 197, 203, 209]		
MCT‐5	SLC16A4 1p13.3	MCT4	(1) Has an Alu insertion in the 3′‐UTR and a shorten C‐terminus (2) 30%–25% similarity of amino acid sequence with MCT1 (3) Orphan transporter	Placenta, heart, egg, liver, muscle, brain, kidney	[74, 75, 119, 197]		
MCT‐6	SLC16A5 17q25.1	MCT5	(1) 30%–25% similarity of amino acid sequence with MCT1 (2) Orphan transporter	Lung, intestine, prostate, muscle, kidney, spleen, heart, brain, pancreas	[73, 74, 75, 119]		
MCT‐7	SLC16A6 17q24.2	MCT6	(1) 30%–25% similarity of amino acid sequence with MCT1 (2) Orphan transporter	Brain, muscle, pancreas	[74, 75, 119]		
MCT‐8	SLC16A2 Xq13.2	MCT7, MRX22DXS128, AHDXPCT	(1) Mutations of MCT8 have been associated with Allan–Herndon–Dudley Syndrome (2) During the review X‐chromosome inactivation discovered (3) Transport undependent Na (4) Essential for transport of thyroid hormone across the blood–brain barrier (5) Facilitated transporter	Most tissues	[73, 75, 119, 197, 210, 211, 212]		
MCT‐9	SLC16A9 10q21.2	YKW1, OXlT‐2C10orf36	Orphan transporter	Testis, ovule, brain, kidney, adrenal, eye, breast	[75, 119]		
MCT‐10 (TAT1)	SLC16A10 6q21‐q22	TAT1PRO0813	(1) Transportation of aromatic amino acids (phenylalanine, tyrosine, and tryptophan) (2) Transport undependent Na and proton (3) 50% similarity with MCT8 (4) Facilitated transporter	Skeletal muscle, heart, kidney, placenta, liver, intestine	[73, 75, 78, 119]		
MCT‐11	SLC16A11 17p13	‐	(1) Orphan transporte	Eye, kidney, lung, skin, breast, ovary	[75, 119]		
MCT‐12	SLC16A12 10q23.3	CJMGCRT2CTRCT47	(1) Orphan transporter	Kidney, testis, eye	[75, 119]		
MCT‐13	SLC16A13 17p13.1	‐	(1) Orphan transporter	Breast, ovary, brain, heart	[75, 119]		
MCT‐14	SLC16A14 2q36.3	‐	(1) Orphan transporter	Eye, pancreas, lung, breast, brain	[75, 119]		

Abbreviations: AICAR, Aminoylamidazole carboxymide‐ribonuclease; AMPK, active protein kinase AMP; CHC, a‐cyano‐4‐hydroxycinnamate; DBDS , 4,4′‐dibenzamidostilbene‐2,2′‐disulphonate; DIDS, 4′‐diisothiocyanostilbene‐2,2′‐disulphonate; pCMBS, p‐chloromercuribenzene sulfonate.

The MCTs have numerous physiological functions. They are often expressed in different tissues and involved in regulating of activities such as gluconeogenesis, lymphocyte activation, spermatogenesis, thyroid hormones metabolism, beta‐pancreatic cells activity, and drug delivery.^63^ In the tumor tissue, the process of transporting and exchanging of lactate among different tumor cells is also carried out MCTs.^8^ The export of lactate from cancer cells is an important factor in maintaining the acidic phenotype and survival of these cells, with considering this assumption, studies showed repression of the MCT‐4 gene reduces tumor growth.^81^, ^82^ Several factors affect the regulation of MCTs expression, for example, it has been shown that hypoxia increases the expression of MCTs.^83^

In patients with (rs1049434) polymorphism, a reduction of 35%–40% in lactate transmission in erythrocytes has been observed.^84^ Two polymorphisms (rs10506398 and rs10506399) in MCT2 have been associated with reduced sperm count and infertility in men.^76^, ^85^ Polymorphism (rs2242206) in MCT‐9 causes lysine to be transformed into threonine at position 258, which is associated with renal overload gout.^86^ A nonsense mutation in the MCT‐12 gene, which causes Q215X amino acid mutation, has been observed in patients with cataract and increased levels of urinary glucose.^87^ In Figure 1, MCT molecules and the effect of them on the process and intracellular molecules in one malignant cell are shown.

The proper expression of MCTs often requires an ancillary protein that for MCT1, 3, and 4 is CD147 (other names are EMMPRIN, OX‐47, HT7, and Basigin) which is MCT chaperone. Ancillary protein for MCT‐2 is Embigin or gp70. These ancillary proteins are a multipurpose glycoprotein of the immunoglobulins family.^8^, ^88^ Upregulation of CD147 has been reported in metastatic breast cancer cells and in partnership with MCT‐4, plays an important role in the invasion of the cancer cells.^89^ CD147 is a highly glycosylated membrane protein belonging to the immunoglobulin family, which in human located at 19p13.3.^90^ The human gene has 10 exons^91^ and encoded 269 amino acids that consist of four types: CD147‐1, CD147‐2, CD147‐3, and CD147‐4. Among these isoforms, CD147‐2 has the most expression and distribution.^92^, ^93^, ^94^, ^95^ This molecule plays a role in regulating of lymphocyte response, cancer metastasis, inducing of MCT, inflammatory reaction, and spermatogenesis.^96^ Interactive proteins with CD147 and their functions have been shown in Table 3.

**Table 3:** Interactive proteins with CD147 and their functions

Protein	Functions	References
Integrins	Adherence, proliferation, migration signal transmission	[97]
CD98	Cell aggregation, skeletal structure	[98, 99]
MCT1, 3, 4	Glycosylation and removal of lactate	[100]
Cavolin‐1	Inhibition of CD147 dimerization and activity, CD147 glycosylation increasing	[101, 102]
S100A	Cell migration	[99]
E‐selectin	Neutrophilic Infiltration and adherence	[103]
CyPB	Cell migration	[104]
yPA	Cell migration, adherence, chemotaxis, Induction of metalloproteinase matrix, Increasing the regulation of metalloproteinase matrix 9, NF‐κB pathway activation	[105, 106, 107]
CD147	Adherence, induction of metalloproteinase matrix, NF‐κB pathway activation	[99, 108]

Abbreviations: CyPA, cyclosporine B; CyPA, cyclosporine A; NF‐κB, nuclear factor kappa‐light‐chain‐enhancer of activated B cells.

Hyaluronan is another protein that interacts with MCTs.^109^ Hyaluronan is an extracellular polysaccharide that is present in most tissues and has an instructive, cell signaling function in addition to its structural role.^110^, ^111^, ^112^, ^113^ The concentration of hyaluronan is more in malignant cells than in normal tissues.^109^, ^114^, ^115^ Hyaluronan binds to various cell surface receptors such as CD44, Rhamm, TLR1/4, Hare and LYVE‐1.^110^, ^111^, ^112^, ^113^ Among these receptors, CD44 is the most known. The binding of this receptor to hyaluronan regulates signaling pathways such as apoptosis and drug resistance to anticancer agents.^109^ Emmprin as an essential ancillary protein for MCTs by stimulating the production of hyaluronan plays an important role in the invasion and division of cancer cells.^112^, ^116^, ^117^ Studies have shown that lactate produced in the glycolysis pathway stimulates the production of hyaluronan and the expression of CD_44_ in fibroblasts and melanoma cells.^53^, ^118^ The interaction of Emmprin‐CD44 is one of the main causes of malignancy and drug resistance in cancer cells.^109^ Several studies have been conducted on the MCTs that the history of major studies and their results from 1999 to 2018 is shown in Table 4.

**Table 4:** The expression of different MCTs in cancer cells

Year	Gene	Result	References
1999	MCT1‐4	They play an important role in the compatibility of cancer cells with glycolysis	[119]
2005	MCT3	Decreased expression of MCT3 in smooth muscle cells and atherosclerosis improvement were seen	[120]
2010	MCT3	MCT3 expression is limited to the retina	[121]
2012	MCT1, 2, and 4	Expression of MCT1 reduces in colon cancer and the MCT2‐4 protein was not detectable In the central nervous system, increasing the expression of MCT1 and 2 was observed In lung and prostate cancers, increasing the expression of all three isoforms was observed	[122]
2013	MCT8	Reducing the expression of this gene plays a role in limiting intrauterine growth of the brain	[123]
2014	MCT1‐4	In lung cancer cells, expression of MCT1 decreases and MCT4 increases. In stromal cells, expression of MCT1 increases and expression of MCT2 and 3 decreases	[124]
2007 and 2014	MCT9	This protein is involved in carnitine (as an antioxidant, with increasing the activity of nitric oxide enzyme, decreasing the activity of the arginase enzyme and the level of ornithine, as well as reducing the side effects of tamoxifen, has an important role in improving the prognosis) transmission	[125, 126]
2014	MCT4	Expression of MCT4 is increased in triple‐negative breast cancer and associates independently with clinical consequence	[127]
2016	MCT1	In glioblastoma cell lines, its effect on cell proliferation and cell migration was investigated Increased expression of MCT1 in cancer stem cells was shown Inhibition of this gene was also proposed as a therapeutic target	[55]
2017	MCT1 and 4	Increased expression of MCT1 and 4 in different breast cancer cell lines, blood and tissues of patients with breast cancer has been seen The study suggested that MCT1 and 4 as can be considered as therapeutic targets	[128]
2017	MCT8	In thyroid cancer, MCT8 expression is reduced Could be considered as a potential thyroid differentiation marker In previous studies, MCT8 expression in the brain, heart, kidney, liver, placenta, and testis was examined	[129]
2018	LDH and MCT1	In ovarian cancer cells, knockdown of SATB1significantly Downregulated both LDH and MCT1 levels Upregulated BRCA1 and BRCA2 levels Promote metastasis, with shorter survival, may reprogram energy metabolism	[130]
2018	LDH‐A and MCT1	miR‐124: Inhibited glycolytic activity of PANC‐1 cells Target MCT1 Decrease the tumor phenotype by increasing the intracellular pH through LDH‐A and HIF‐1α Overexpression of miR‐124 and silencing of MCT1 significantly inhibited growth of tumor	[131]

Abbreviation: HIF, hypoxia‐inducible factor.

### 3.4 Diagnostic and therapeutic applications of metabolic changes in the cancer cells

Cancer is a disease that is known to alter cellular metabolism; therefore, metabolomics can play a major role in the early detection and diagnosis of cancer and in the evaluation of medical interventions and therapies to cancer.^132^ It has been established that aerobic glycolysis increases in cancer and this is known as the “Warburg effect.”^133^ The ultimate goal of most metabolomics cancer studies is to discover cancer‐specific diagnostic, prognostic, or predictive biomarkers for a patient. Metabolomics research is being used to discover diagnostic cancer biomarkers in the clinic, to better understand its complex heterogeneous nature, to discover pathways involved in cancer that could be used for new targets and to monitor metabolic biomarkers during therapeutic intervention.^134^ These metabolomics approaches may also provide clues to personalized cancer treatments by providing useful information to the clinician about the cancer patient's response to medical interventions. Therapeutics in oncology is touching toward the use of medications that specifically target abnormal pathways involved in growth, proliferation, and metastases. Biomarkers are being increasingly applied in the early clinical development of such agents to identify, validate, and optimize therapeutic targets and agents; determine and confirm the mechanism of medications action, as a pharmacodynamic endpoint; and in predicting or monitoring responsiveness to treatment, toxicity, and resistance.^135^ Current examples of using metabolomics in developmental therapeutics are with tyrosine kinase inhibitors, proapoptotic agents, and heat shock protein inhibitors.^136^, ^137^, ^138^ Numerous metabolic inhibitors have been reported for cancer treatment both in preclinical studies and in clinical trials, included amino acid metabolisms inhibitor, lipid metabolisms inhibitor, glutamines inhibitor, coenzyme/nucleotide inhibitor, mitochondrial inhibitors, and transporter inhibitors. The development of metabolic inhibitors has been well addressed in a recently published review paper.^139^

**Figure 2:**
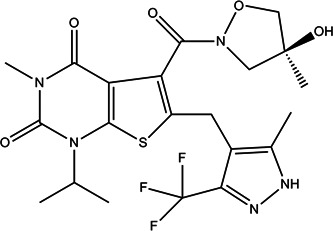
Structures of AZD3965; selective inhibitor of MCT‐1, currently under clinical investigation

AZD3965 (Figure 2) is a first‐in‐class, strong (K1 = 1.6 nM), orally bioavailable, MCT‐1 selective inhibitor. As cancer cells exhibit an elevated dependence on glycolysis lead to lactic acid formation, they up‐regulate MCTs as a protective system to transport lactate and inhibit its intracellular accumulation.^140^ Significant antiproliferative effects of AZD3965 have been reported in some lymphoma cell lines as well as small‐cell lung tumor systems.^141^ A pharmacodynamics indicator investigation has been shown that AZD3965 therapy lead to reduced lipid metabolisms including reduced choline concentration. Decreased choline levels has been attributed to reduced choline kinase A because of elevated intracellular levels of lactate in cancer cells.^141^ AZD3965‐treated cancers show an increased frequency of dendritic cells and natural killer cells.^142^ Currently, AZD3965 is being evaluated in a phase I dose‐ranging study.^139^

## 4 CONCLUSION

Changed energy metabolism is emerged as one of the very important cancer biochemical fingerprint which could be introduced as a cancer hallmark. These metabolic events are characterized by preferential dependence on glycolysis for the production of energy in an oxygen‐independent condition. Glycolysis is one of the major underlying energy‐related processes, which play critical roles in the initiation, and progression of all malignancies. Pyruvate and lactate are the main products of glycolysis. Given that, some cellular proteins (i.e., LDH and MCTs) involved in the production and transport of lactate. Considering the vital role of LDH and MCTs in the metabolism of cancer cells, these molecules can be investigated as a target for treatment or as markers in cancer diagnosis in future studies. Mounting evidence confirmed that these molecules could contribute to escape cancer cells from the immune system and promote the expansion of tumor cells under distinct conditions.

## AUTHOR CONTRIBUTIONS

**Hamed Hatami**: Investigation; methodology; software; validation; writing – original draft; writing – review and editing. **Atefe Sajedi**: Data curation; investigation; writing – original draft. **Seyed Mostafa Mir**: Conceptualization; investigation; methodology; software; supervision; writing – original draft; writing – review and editing. **Mohammad Yousef Memar**: Conceptualization; methodology; software; supervision; validation; writing – review and editing.

## TRANSPARENCY STATEMENT

The lead author Seyed Mostafa Mir, Mohammad Yousef Memar affirms that this manuscript is an honest, accurate, and transparent account of the study being reported; that no important aspects of the study have been omitted; and that any discrepancies from the study as planned (and, if relevant, registered) have been explained.

## Supporting information

Supplementary information.

## Data Availability

Seyed Mostafa Mir and Mohammad Yousef Memar had full access to all the data in this study and takes complete responsibility for the integrity of the data and the accuracy of the data analysis.
