# Supplementary material for: Importance of lactate dehydrogenase (LDH) and monocarboxylate transporters (MCTs) in cancer cells
Source: Health Sci Rep. 2022 Dec 21;6(1):e996. doi: 10.1002/hsr2.996 (PMC9768844; doi:10.1002/hsr2.996)
Supplement: Supplementary file 1 — Supplementary information. [file HSR2-6-e996-s001.docx]

Flow chart of the literature selection process for the Tables in the present articles

-Excluded studies

-Review and congress abstract

-Studies not reporting

Excluded Studies

Title and abstract screening

Studies detected by the initial screening of databases: n=

Google scholar

PubMed

Included studies

Full text

Duplication

Scopus
